# Insufficient HtrA2 causes meiotic defects in aging germinal vesicle oocytes

**DOI:** 10.1186/s12958-022-01048-4

**Published:** 2022-12-20

**Authors:** Min Gao, Yanling Qiu, Tianqi Cao, Dungao Li, Jingwen Wang, Yiren Jiao, Zhiyun Chen, Junjiu Huang

**Affiliations:** 1 MOE Key Laboratory of Gene Function and Regulation, State Key Laboratory of Biocontrol, School of Life Sciences, Sun Yat-sen University, Guangzhou, 510275 China; 2 Guangdong Laboratory for Lingnan Modern Agriculture, Guangzhou, 51000 China; 3 Key Laboratory of Reproductive Medicine of Guangdong Province, School of Life Sciences and the First Affiliated Hospital, Sun Yat-sen University, Guangzhou, 510275 China; 4 The Reproduction Medicine Center of Hui Zhou Municipal Central Hospital, Huizhou, 516001 China

**Keywords:** Aging, HtrA2, Germinal vesicle oocyte, Maturation, Meiosis

## Abstract

**Background:**

High-temperature requirement protease A2 (HtrA2/Omi) is a mitochondrial chaperone that is highly conserved from bacteria to humans. It plays an important role in mitochondrial homeostasis and apoptosis. In this study, we investigated the role of HtrA2 in mouse oocyte maturation.

**Methods:**

The role of HtrA2 in mouse oocyte maturation was investigated by employing knockdown (KD) or overexpression (OE) of HtrA2 in young or old germinal vesicle (GV) oocytes. We employed immunoblotting, immunostaining, fluorescent intensity quantification to test the HtrA2 knockdown on the GV oocyte maturation progression, spindle assembly checkpoint, mitochondrial distribution, spindle organization, chromosome alignment, actin polymerization, DNA damage and chromosome numbers and acetylated tubulin levels.

**Results:**

We observed a significant reduction in HtrA2 protein levels in aging germinal vesicle (GV) oocytes. Young oocytes with low levels of HtrA2 due to siRNA knockdown were unable to complete meiosis and were partially blocked at metaphase I (MI). They also displayed significantly more BubR1 on kinetochores, indicating that the spindle assembly checkpoint was triggered at MI. Extrusion of the first polar body (Pb1) was significantly less frequent and oocytes with large polar bodies were observed when HtrA2 was depleted. In addition, HtrA2 knockdown induced meiotic spindle/chromosome disorganization, leading to aneuploidy at metaphase II (MII), possibly due to the elevated level of acetylated tubulin. Importantly, overexpression of HtrA2 partially rescued spindle/chromosome disorganization and reduced the rate of aneuploidy in aging GV oocytes.

**Conclusions:**

Collectively, our data suggest that HtrA2 is a key regulator of oocyte maturation, and its deficiency with age appears to contribute to reproduction failure in females.

**Supplementary Information:**

The online version contains supplementary material available at 10.1186/s12958-022-01048-4.

## Introduction

In mammals, growing oocytes store large amounts of maternal mRNAs and proteins that alter the functional capabilities, dynamics, and biophysical properties of microtubules [1, 2]. Aged germinal vesicle (GV) stage oocytes show various abnormalities related to the cytoskeleton, including aberrant gene expression, disrupted microtubule assembly and stability, activation of spindle assembly checkpoints (SACs), hyperacetylation of α-tubulin, and aneuploidy [3]. Quantitative proteomic analysis using stable isotope labeling by amino acids in cell culture and mass spectrometry has identified hundreds of differentially expressed proteins between aged and young mouse oocytes [4]. For example, Mphosph10 and Trim71, which are involved in protein modification, become significantly more abundant with maternal age. Instead, proteins related to cytoskeleton assembly and chromosome segregation, such as Tuba3A and Myl1, become less abundant. High-temperature requirement protease A2 (HtrA2), a mitochondrial chaperone, is also downregulated in the oocytes of aged mice [4]; however, its role in oocyte maturation remains unclear.

The high-temperature requirement protease A (HtrA) family includes oligomeric serine proteases, which are highly conserved from bacteria to humans. They participate in a variety of biological and pathophysiological processes [5, 6]. Mammals possess four HtrA family members: HtrA1, HtrA2/Omi, HtrA3, and HtrA4 [6]. The abnormal expression of HtrA members may cause adverse pregnancy outcomes [7]. HtrA1, HtrA3, and HtrA4 include an insulin-like growth factor binding domain crucial for placental and fetal growth during gestation. HtrA2, which does not contain such a domain, participates in cell apoptosis to ensure the physiological turnover of trophoblasts and in this way affects pregnancy outcome [8–10]. HtrA2 is located in the mitochondrial intermembrane space where it contributes to mitochondrial homeostasis and protects against cellular stress under normal physiological conditions [11, 12]. Once apoptosis is triggered, HtrA2 is released into the cytosol, where it binds to and degrades apoptotic factors, such as XIAP and CIAP1/2 [13–16]. Loss of HtrA2/Omi activity in S276C missense mutant mice or gene knockout in non-neuronal tissues of adult mice results in accelerated aging phenotypes, such as premature weight loss, hair loss, reduced fertility, heart enlargement, increased autophagy, and death by 12–17 months. The offspring of such mice die prematurely owing to neurodegeneration 30–40 days after birth [17–20].

Several cytoskeletal proteins, including α- or β-tubulin, are believed to be HtrA2 substrates based on a proteome-wide screen of Jurkat cell lysates [21]. Spindle organization depends on the rapid reorganization of dynamic microtubules composed of α- or β-tubulin dimers [22]. In aged meiotic oocytes, disruption of these dynamics leads to reduced microtubule stability, resulting in abnormal spindle organization [23, 24]. Inefficient control of the spindle/chromosomes in this process produces aneuploid oocytes, which is the main reason for infertility or birth defects in mammals [25, 26]. However, the underlying causes of age-related meiotic defects during mouse oocyte maturation remain unclear.

In this study, we aimed to understand the role of HtrA2 in aging oocytes. Based on knockdown and overexpression of HtrA2, we report that HtrA2 plays an essential role in regulating oocyte maturation. Remarkably, HtrA2 overexpression attenuated the meiotic defects in aged oocytes as it counteracted the natural age-related decline in HtrA2.

## Results

### Insufficient HtrA2 in aging mouse oocytes

**Fig. 1:**
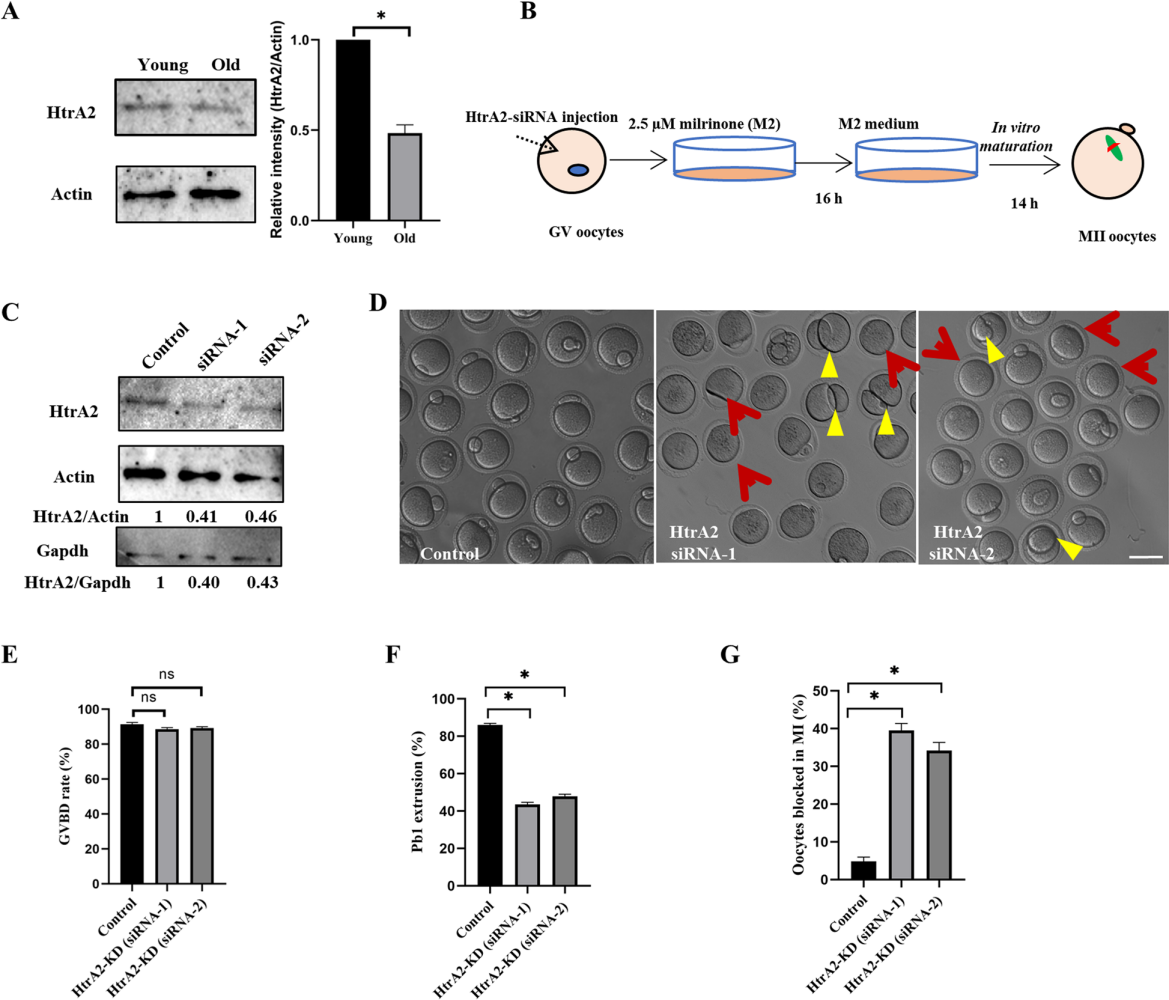
Reduced expression of HtrA2 in aged oocytes. **A** Representative western blot showing HtrA2 expression in oocytes from young and old mice. The relative amount of HtrA2 in old mice was estimated based on the level of actin, which was used as control. Statistical analysis of western blotting in GV oocytes from aged or young mice. Normalized band intensities: HtrA2/Actin. HtrA2 protein levels were significantly lower in oocytes obtained from old mice compared to young mice. Data are expressed as the mean ± SEM of three independent experiments. *, *p* < 0.05. **B** Diagram summarizing the HtrA2-KD approach using siRNA microinjection. **C** Western blotting verification of endogenous HtrA2 knockdown using two different siRNAs and 100 fully grown GV-stage oocytes per lane. The relative amount of HtrA2 in HtrA2-KD oocytes was estimated based on the level of actin and/or glyceraldehyde 3-phosphate dehydrogenase (GAPDH). **D** Phase-contrast images of control and HtrA2 siRNA injected oocytes. Yellow arrowheads point to oocytes with symmetrical division or a large polar body. Red arrows point to oocytes with no polar body. Scale bar: 80 μm. **E–G** Quantitative analysis of the GVBD rate, extrusion frequency of the first polar body (Pb1; control *n* = 341; siRNA-1 *n* = 255; siRNA-2 *n* = 298), and percentage of oocytes arrested at metaphase I (control *n* = 164; siRNA-1 *n* = 167; siRNA-2 *n* = 153) in control and HtrA2-KD oocytes. Data are expressed as the mean ± SEM from three independent experiments. *, *p* < 0.05. ns, no significant difference

To determine the level of HtrA2 in GV oocytes from aged or young female mice, we analyzed the corresponding lysates by western blotting. We found significantly decreased HtrA2 protein levels in aged GV stage oocytes (Fig. 1A). Based on this result, we designed and microinjected two HtrA2 siRNAs into young GV stage oocytes to explore how the reduction in HtrA2 affected their maturation in vitro (Fig. 1B). Both siRNA-1 and siRNA-2 reduced about 50% the expression of HtrA2 (Fig. 1C), which affected the in vitro maturation of oocytes (Fig. 1D). HtrA2 knockdown (KD) did not diminish the germinal vesicle breakdown (GVBD) ratio (Fig. 1E); however, it more than halved the frequency of extrusion of the first polar body (siRNA-1: 43.56% ± 1.17%; siRNA-2: 47.94% ± 1.07%) compared to the control group (86.01% ± 0.98%) (Fig. 1F). A higher frequency of symmetrical division was also observed in HtrA2-KD oocytes. Specifically, 34–39.5% HtrA2-KD young oocytes were arrested at MI, which was notably higher than in control oocytes (4.85% ± 1.11%) (Fig. 1G). Overall, these data suggested that knockdown of HtrA2 blocked the maturation of young oocytes in vitro. Since siRNA-1 showed better knockdown efficiency, we used it in the subsequent knockdown experiments.

### HtrA2 knockdown activates the SAC in young mouse oocytes

**Fig. 2:**
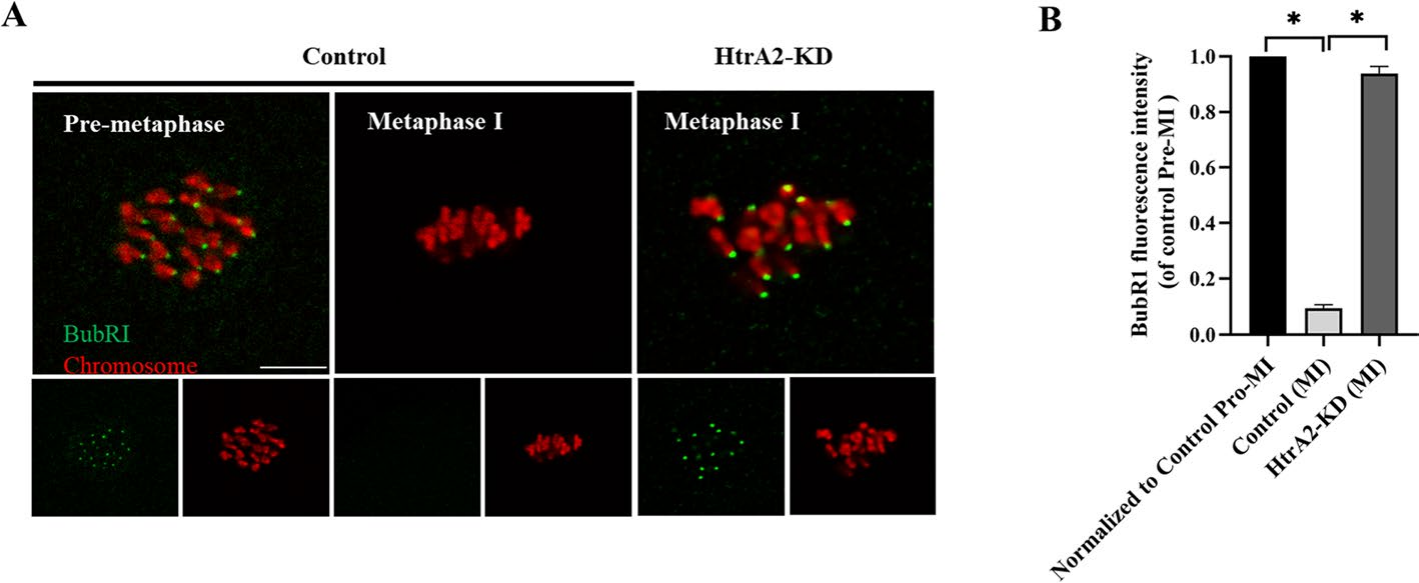
HtrA2 knockdown activates the SAC in young oocytes. **A** Control and HtrA2-KD oocytes were stained with anti-BubR1 antibody (green) and counterstained with propidium iodide to visualize the chromosomes (red). Representative images of pre-metaphase I and metaphase I oocytes are shown. **B** Quantitative analysis of BubR1 fluorescence intensity in control (*n* = 25) and HtrA2-KD (*n* = 30) oocytes. Data are expressed as the mean ± SEM of three independent experiments. *, *p* < 0.05. Scale bar: 5 μm. HtrA2-KD, use siRNA-1

Given that decreased HtrA2 expression impeded extrusion of the first polar body, we hypothesized that HtrA2 might be involved in spindle organization or migration during meiosis. During this process, the spindle assembly checkpoint (SAC) acts as a safety mechanism, which holds the oocyte in the metaphase-to-anaphase transition of meiotic phase I until all chromosomes are aligned and microtubules have been correctly attached [27]. Given the arrest in MI of HtrA2-KD young oocytes, we hypothesized potential activation of the SAC. The mitotic checkpoint serine/threonine kinase BubR1 is an important component of the SAC [28]. Generally, BubR1 disappears at MI, when kinetochores become properly attached to microtubules. Consistently, BubR1was present on the kinetochores in HtrA2-KD young oocytes instead of control oocytes at metaphase I stage, implying SAC activation (Fig. 2A, B). Collectively, these results suggested that HtrA2-KD triggered SAC activation during meiosis.

### HtrA2 knockdown causes cytoskeletal disorganization in young mouse oocytes

**Fig. 3:**
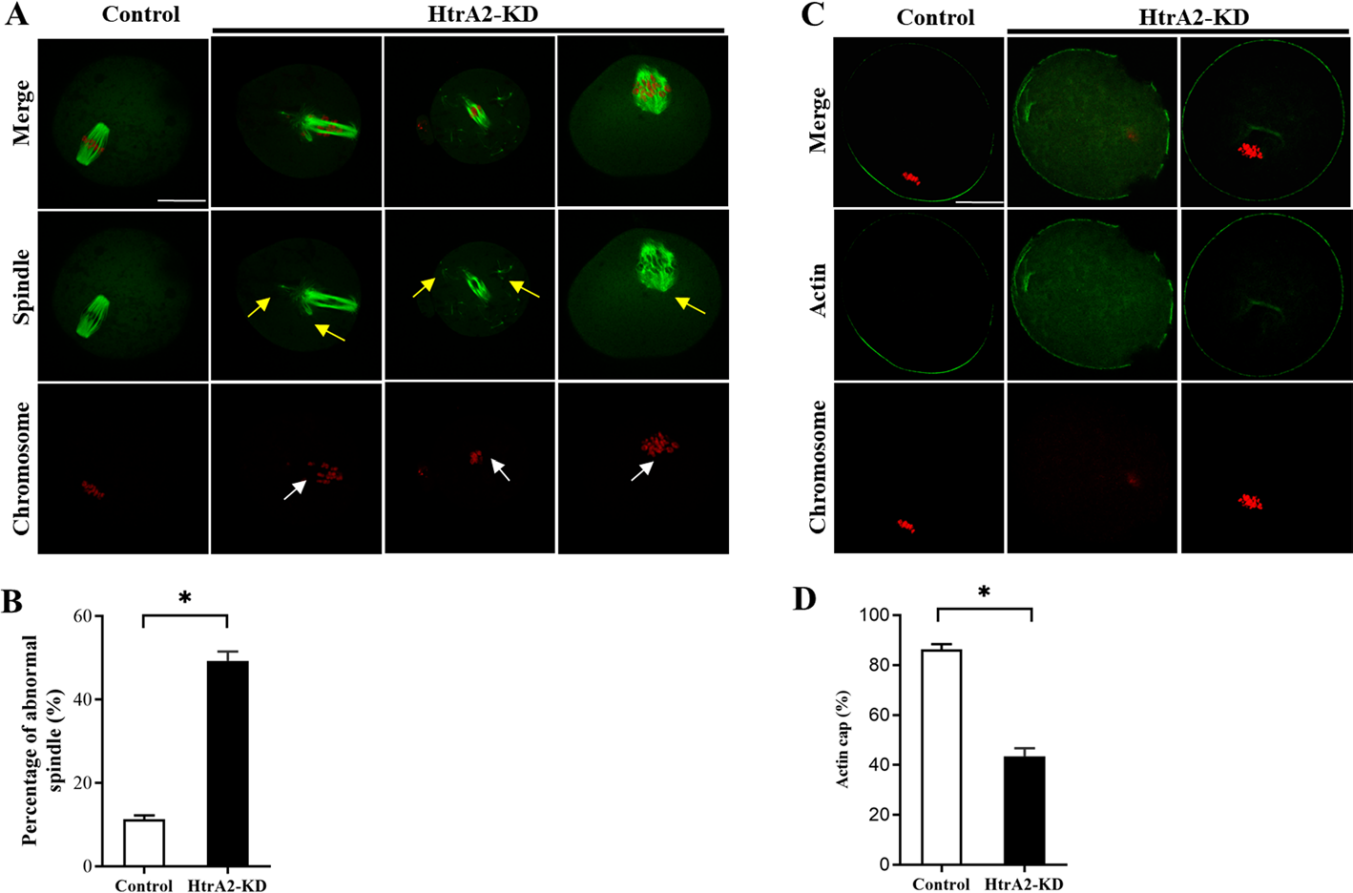
HtrA2 knockdown disrupts cytoskeletal organization during oocyte meiosis. **a **Control and HtrA2-KD oocytes were stained with an anti-α-tubulin antibody to detect the spindle (green) and counterstained with propidium iodide to visualize the chromosomes (red). Control oocytes (*n* = 83) showed a typical barrel-shaped spindle. Disorganized spindles (yellow arrows) and chromosomes (white arrows) were readily observed in HtrA2-KD oocytes (*n* = 65). Scale bar: 25 μm. **b** Quantification of control and HtrA2-KD oocytes with spindle defects. **c **Metaphase II oocytes were labeled with phalloidin to visualize actin (green) and counterstained with propidium iodide for chromosomes (red). **d **Quantification of control (*n* = 43) and HtrA2-KD (*n* = 61) oocytes with normal actin cap formation. Data are expressed as the mean ± SEM of three independent experiments. *, p < 0.05. HtrA2-KD, use siRNA-1

The SAC is desensitized during oocyte meiosis, permitting chromosomal segregation in the presence of several chromosomal misarrangements [27]. Even though the SAC was induced during meiosis, almost 44% of HtrA2-KD oocytes extruded the first polar body (Fig. 1F). Because oocyte maturation depends on the coordination between spindle and actin dynamics [29], we detected MII oocyte spindles using an anti-α-tubulin antibody. Nearly 89% of MII oocytes in the control group exhibited typical barrel-shaped spindles and well-aligned chromosomes on the metaphase plate. In contrast, about 49% of spindles appeared disrupted in HtrA2-KD young oocytes (Fig. 3A,B). Actin polymerization participates in spindle migration, actin cap formation, and polar body extrusion [30]. To determine whether HtrA2-KD affected actin polymerization, actin was labeled with phalloidin (Fig. 3C). As shown in Fig. 3D, actin caps were visible in approximately 86 and 43% of control and HtrA2-KD young oocytes, respectively. Mitochondria has been considered as an important factor in determining the quality of mammalian oocytes. The mitochondrial energy metabolism, such as ATP and reactive oxygen species (ROS), are considered as necessary factors for spindle organization and chromosome alignment during oocyte meiotic progresses [23] . ATP provides energy for oocyte cytoskeletal organization, and generated ROS as the byproduct [31]. Excessive level of ROS induces DNA damage, apoptosis or aging [32]. Thus, we evaluated whether HtrA2-KD influenced cytoskeletal disorganization was related to mitochondrial distribution during mouse oocytes maturation. MII stage oocytes were labeled with Mito Tracker for mitochondrial dynamics. Mitochondria normally gathered around the spindle. In striking contrast, homogeneous distribution of mitochondria in the cytoplasm were observed in HtrA2-KD oocytes (Supplement Fig. 1 A, B). Therefore, we speculated that HtrA2 knockdown impaired cytoskeletal organization due to mitochondria disfunction.

### HtrA2 knockdown promotes aneuploidy in young mouse oocytes

**Fig. 4:**
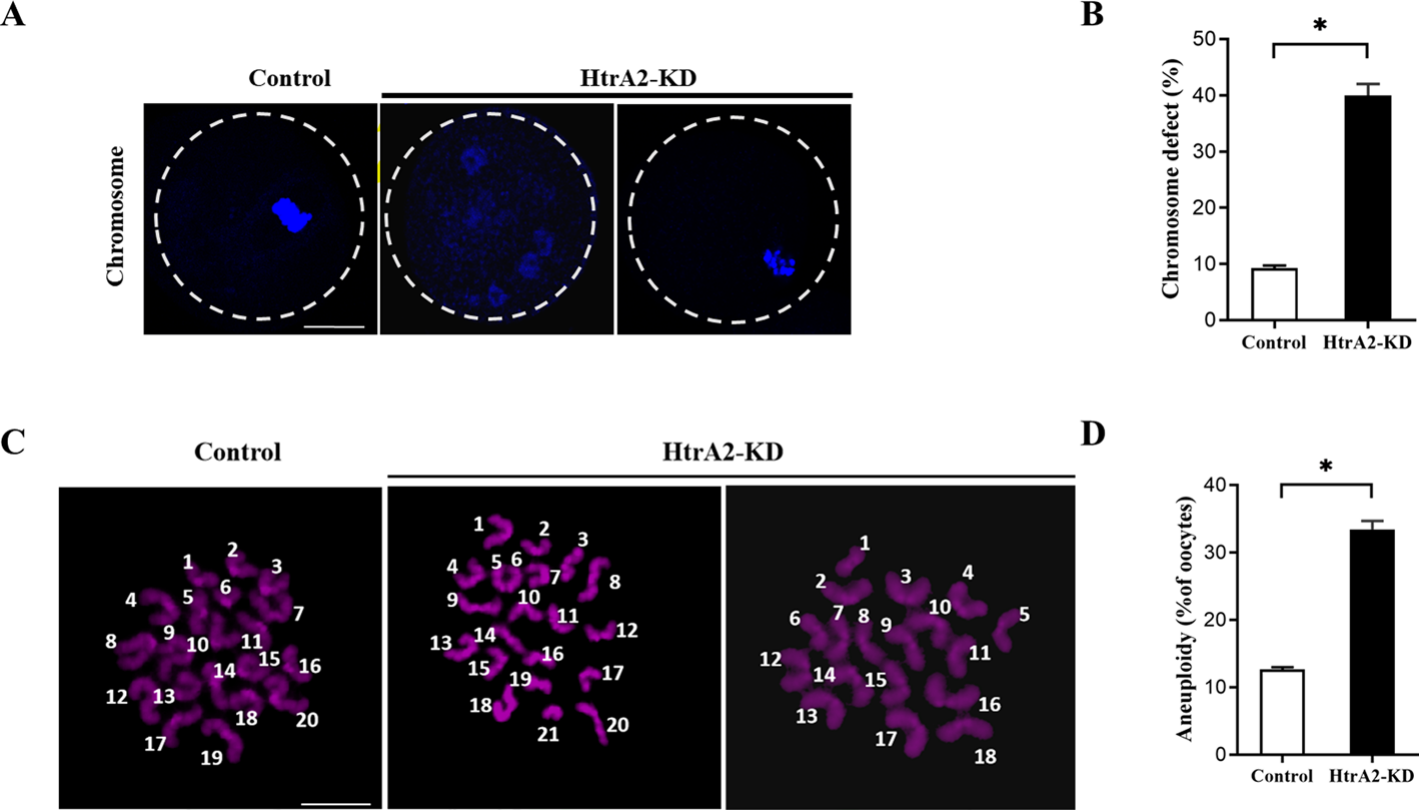
Knockdown of HtrA2 augments the incidence of aneuploidy in mouse oocytes. (A) Control and HtrA2-KD metaphase II oocytes were labeled with Hoechst 33342 to visualize the chromosomes (blue). Representative confocal images are shown. Scale bar: 10 μm. (B) Quantitative analysis of chromosome defects in control (*n* = 62) and HtrA2-KD (*n* = 119) oocytes. (C) Chromosome spreading of control and HtrA2-KD metaphase II oocytes. Chromosomes were stained with Hoechst 33342 (purple). (D) Quantification of aneuploidy in control (*n* = 43) and HtrA2-KD (*n* = 45) oocytes. Data are expressed as the mean ± SEM of three independent experiments. *, p < 0.05. HtrA2-KD, use siRNA-1

Chromosome segregation errors are driven by spindle disorganization during oocyte meiosis [33]. About 9% of control MII oocytes presented misaligned chromosomes. And the ratio was markedly higher (40% ± 2.0%) in HtrA2-KD oocytes (Fig. 4A, B). To determine whether spindle and chromosome abnormalities in HtrA2-KD young oocytes led to aneuploid oocytes, the karyotype of MII oocytes was analyzed by chromosome spreading. We found that the number of chromosomes either increased or decreased in HtrA2-KD oocytes (Fig. 4C). Quantitative analysis (Fig. 4D) revealed that the loss of HtrA2 resulted in a 2.75-fold increase in the incidence of aneuploidy compared to control oocytes. These observations pointed to an increased likelihood of aneuploid eggs when HtrA2 is suppressed.

### HtrA2-KD young oocytes present an increased level of α-tubulin acetylation

**Fig. 5:**
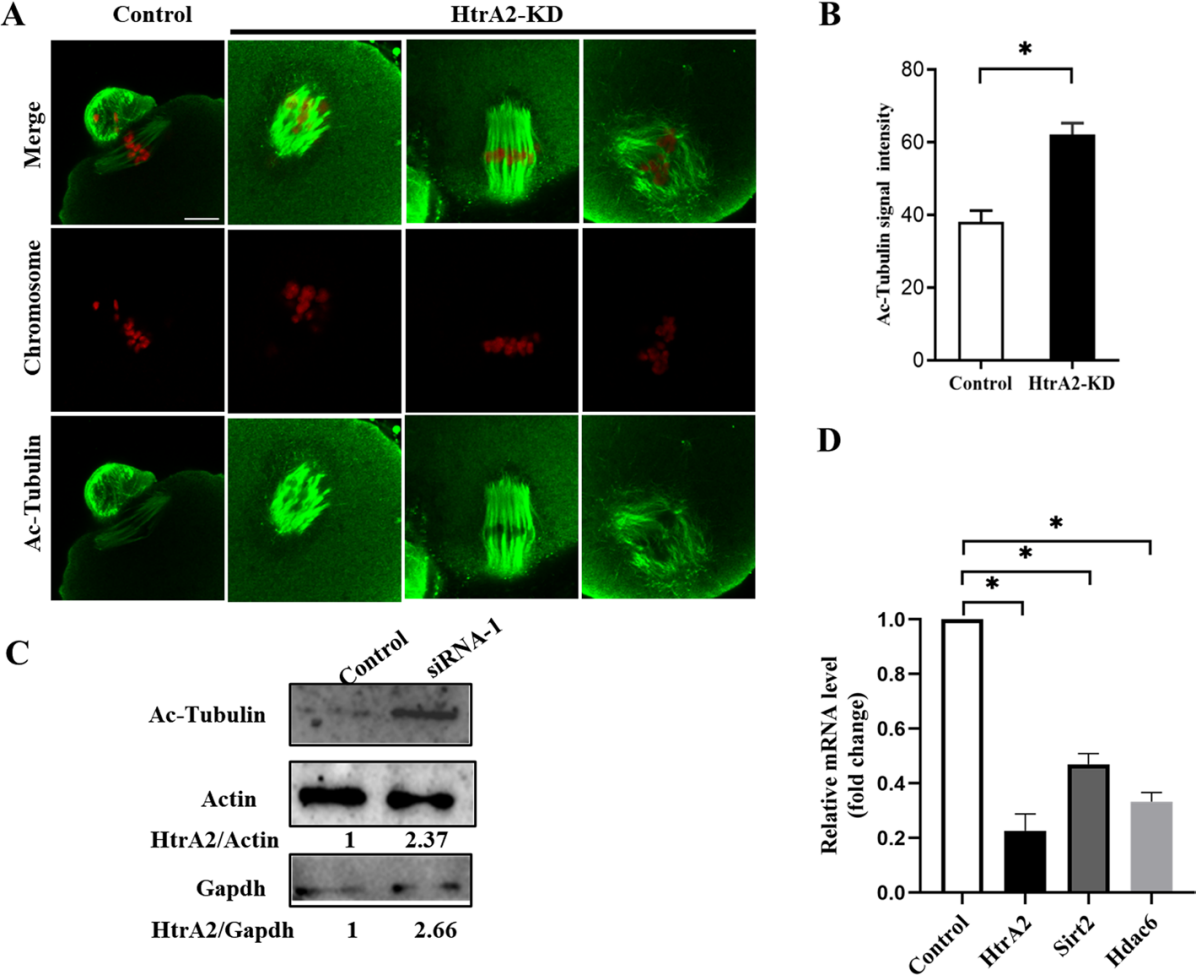
Hyperacetylation of α- tubulin in HtrA2-KD oocytes. (A) Metaphase II oocytes were labeled with an anti-acetylated α-tubulin antibody (green) and counterstained with propidium iodide to visualize the chromosomes (red). Representative images of control and HtrA2-KD oocytes are shown. Scale bar: 5 μm. (B) Quantification of fluorescence intensity from acetylated tubulin in control (*n* = 10) and HtrA2-KD (*n* = 34) oocytes. (C) Representative western blots showing the expression of acetylated tubulin in oocytes from control and HtrA2-KD mice (*n* = 100 per group). The relative amount of HtrA2 was estimated based on the level of actin and GAPDH. (D) Quantitative RT-PCR analysis of gene expression in control and HtrA2-KD oocytes. *, p < 0.05. HtrA2-KD, use siRNA-1

Mounting evidence has highlighted the importance of α-tubulin acetylation in stabilizing microtubules [34–36]. Recently, α-tubulin acetylation levels were reported to be markedly increased in aged oocytes during meiosis [35]. To explore the potential mechanism of spindle and chromosome disorganization in oocytes, we examined α-tubulin acetylation during meiosis (Fig. 5A). The level of acetylated-tubulin was significantly increased in HtrA2-KD young oocytes (signal intensity: 62.09 ± 3.17) compared to controls (signal intensity: 38.14 ± 3.08) (Fig. 5B). This trend was confirmed by western blot analysis (Fig. 5C). Next, we assessed how HtrA2 regulated the function of deacetylases and modulated tubulin acetylation. Quantitative RT-PCR revealed significantly decreased expression of the deacetylases Sirt2 (47%) and Hdac6 (33%) in HtrA2-KD oocytes (Fig. 5D) compared to the control group. These results demonstrate that HtrA2 regulates microtubule stability by modulating the action of deacetylation-related enzymes.

### HtrA2 overexpression compensates for meiotic defects in aging oocytes

**Fig. 6:**
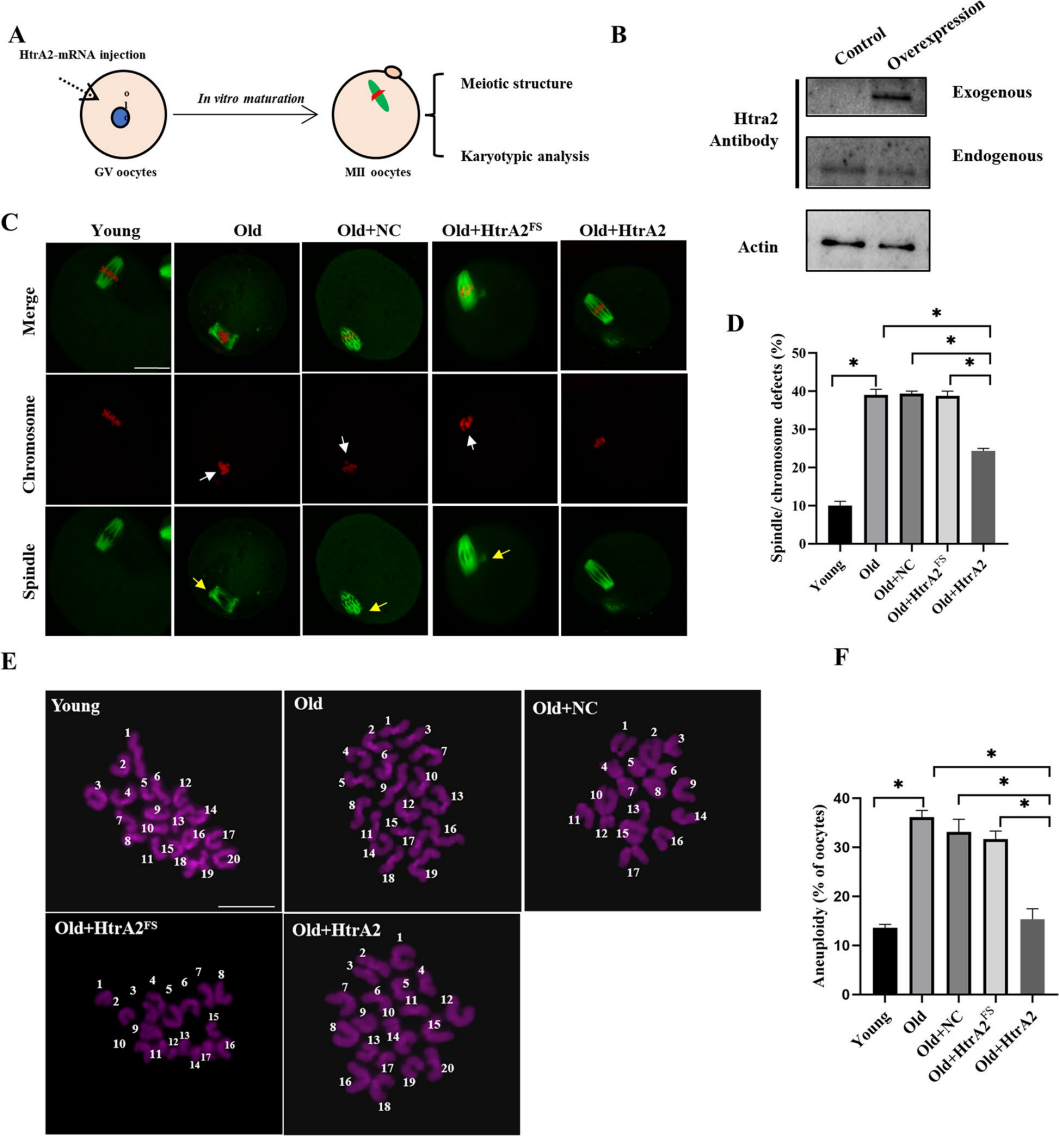
HtrA2 overexpression improves oocyte quality in old mice. **A** Schematic representation of the experimental procedure used to determine whether HtrA2 overexpression could rescue the meiotic defects in oocytes from old mice. **B** Western blot analysis confirming successful overexpression of exogenous HtrA2 using with an anti-HtrA2 antibody (*n* = 85 per group). In the overexpression group, approximately 10 pl HtrA2-EGFP mRNA was injected into young GV oocyte. An equal amount of nuclease-free water was injected into young oocytes as a control. (C) Control, Old, Old + NC (RNase-free water), Old+HtrA2^FS^ (encoding frame shifted HtrA2 mRNA) and Old+HtrA2-mRNA (remove EGFP) oocytes were stained with an anti-α-tubulin antibody to visualize the spindle (green) and counterstained with propidium iodide to visualize the chromosomes (red). Arrows point to disorganized spindle (yellow arrows) and misaligned chromosomes (white arrows). Scale bar: 25 μm. **D** Quantification of Young (*n* = 60), Old (*n* = 45), Old+NC (*n* = 36), Old+HtrA2^FS^ (*n* = 20), and Old+HtrA2-mRNA (*n* = 60) oocytes with spindle/chromosome defects. **E** Chromosome spreading of Young, Old, Old+NC, Old+HtrA2^FS^ and Old+HtrA2-mRNA oocytes. Chromosomes were stained with Hoechst 33342 (purple). Scale bar: 10 μm. **F** Quantification of aneuploidy in Young (*n* = 28), Old (*n* = 33), Old+NC (*n* = 24), Old+HtrA2^FS^ (*n* = 13), and Old+HtrA2-mRNA (*n* = 32) oocytes. Data are expressed as the mean ± SEM of three independent experiments. *, *p* < 0.05. HtrA2-KD, use siRNA-1

Finally, we investigated whether exogenous HtrA2 could, at least in part, rescue meiotic defects in aging oocytes (Fig. 6A). First, we confirmed whether exogenous HtrA2 was successfully overexpressed in oocyte. Overexpression group was injected about 10 pl HtrA2-EGFP mRNA solution into young GV oocytes cytoplasm. The same amount of nuclease-free water was injected as a control group. Immunoblotting shown that exogenous HtrA2 was efficiently expressed in GV oocytes (Fig. 6B). Then, HtrA2 mRNA (remove EGFP), RNase-free water (negative control, NC), or HtrA2^FS^ mRNA (encoding frame shifted HtrA2) was microinjected into aged GV oocytes individually, and the spindles of MII oocytes were checked after in vitro maturation (Fig. 6C). More than 39% spindle/chromosome disorganization was detected in nature aged oocytes comparing with young oocytes; however, this phenotype was rescued in part via HtrA2 overexpression but not the dysfunctional HtrA2^FS^ variant (Fig. 6D). To evaluate the effect of HtrA2 overexpression on the karyotype of aged oocytes, we performed chromosome spreading (Fig. 6E) and detected nearly 36% aneuploid oocytes in aged mice, but only almost 15.33% in those overexpressing HtrA2 (Fig. 6F). Overall, these results suggest that loss of HtrA2 contributed significantly to meiotic defects in aging oocytes.

## Discussion

Aging is associated with suboptimal reproductive performance in women. In mammals, maternal age can lead to a decline in oocyte quality and cause infertility or birth defects [37, 38]. However, the potential causes of maturation defects in aged oocytes are not completely understood. Here, we detected a decrease in HtrA2 expression in aging mouse oocytes. To ascertain the role of HtrA2 in aging, we knocked down HtrA2 in young oocytes to mimic HtrA2-depleted aged mouse oocytes. Our results demonstrated that HtrA2 is an important factor in the regulation of maternal age-induced meiotic deficiency in mouse oocytes.

In meiotic oocytes, the presence of BubR1, Mad1 and Mad2 on kinetochores at MI are indicators of SAC activation [39, 40]. Advanced maternal age lowers the level of Hdac3 in oocytes, causing microtubules to detach from kinetochores, which in turn activates the SAC and contributes to meiotic defects and arrested oocyte maturation [35]. Our results showed markedly increased levels of BubR1 on the kinetochores in HtrA2-KD young oocytes at MI, which activated the SAC and caused oocytes to be partly arrested at MI. Notably, the SAC cannot completely block the onset of anaphase, even with several chromosome mis-segregations [27]. We also confirmed that HtrA2 knockdown not only blocked MI, but it also led to abnormal polar body extrusion during oocyte maturation. Previous reports have demonstrated a close relationship between actin assembly, spindle migration, and oocyte maturation [41–43]. Therefore, we presumed that HtrA2 might play an essential role in modulating microtubule organization. In line with this hypothesis, we show that HtrA2-KD disrupted spindle organization and actin cap formation during mouse oocyte maturation. In mammals, inefficient control of spindle and chromosomal organization during oocyte meiosis results in aneuploidy, which may lead to infertility, miscarriage or birth defects [25, 26, 44]. Aneuploid embryos account for at least 50% of pregnancies in women nearing the end of their reproductive lifespan [45]. Here, chromosome disorganization in the form of chromosomes scattered around the metaphase plate was frequently detected in HtrA2-KD young oocytes. We also found that HtrA2 knockdown significantly increased the rate of aneuploidy in young mouse oocytes. The incidence of aneuploidy increases strikingly with female age, and represents a major risk factor for birth defects or pregnancy loss. Our results indicate that HtrA2 knockdown disrupts cytoskeletal organization and actin assembly, thereby causing aneuploidy in oocytes during meiosis.

Microtubules are regulated through various post-translational modifications such as acetylation, tyrosination, and polyglutamylation [46]. In mammalian oocytes, acetylated α-tubulin is considered a marker of stable microtubules ready for spindle organization [47]. Notably, tubulin hyperacetylation was found in aged oocytes, and the expression of some deacetylases was markedly decreased [35]. Here, we observed a significant increase in α-tubulin acetylation in HtrA2-KD young oocytes during meiosis. Based on these findings, we investigated how HtrA2 modulated α-tubulin acetylation. Several deacetylases have been reported to participate in α-tubulin acetylation in somatic cells and oocytes [24, 35, 48–50]. The histone deacetylases Sirt2 and Hdac6 are widely used to modulate tubulin acetylation and regulate microtubule stability during meiosis [43, 48, 51, 52]. During mouse oocyte maturation, Sirt2 modulates the acetylation of histone, H4K16, and α-tubulin and influences microtubule dynamics and kinetochore function [53]. Qiu et al. found that specific depletion of Sirt2 disrupted spindle and chromosome organization and compromised kinetochore-microtubule attachments in mouse oocytes [24]. And HDAC6 inhibitors disrupt spindle migration, actin cap formation, and asymmetric division during oocyte maturation [54]. It may because HDAC6 inhibition can markedly increase tubulin acetylation, which could be related to the disruption of meiotic apparatus assembly [55]. Consistent with these observations, our data indicated that HtrA2 may regulate microtubule stability by affecting both Sirt2 and Hdac6 expression. Intriguingly, α-tubulin was identified as the HtrA2 substrate in a proteome-wide screen of Jurkat cell lysates [21]. Moreover, several spindle-related proteins regulate oocyte mitosis or the meiotic process by affecting microtubule acetylation [56]. For example, Rbm14 interacts with α-tubulin and regulates α-tubulin acetylation of microtubules to affect spindle and chromosome organization in mouse oocytes during meiosis [57]. Therefore, we cannot rule out that HtrA2 might interact with the spindle and regulate the meiotic process by affecting microtubule acetylation. Notably, overexpression of HtrA2 partially rescued the defective aging phenotypes, demonstrating the positive effect of HtrA2 on oocyte quality in aging mice.

## Conclusions

Our results suggest that HtrA2 knockdown disrupts cytoskeletal organization during mouse oocyte maturation probably due to α-tubulin hyperacetylation, thereby producing aneuploid oocytes. The present study may offer practical clues for mouse oocyte quality assessment, especially in aging oocytes.

## Materials and methods

All chemicals were purchased from Sigma Chemical Co., unless otherwise indicated.

### Animal care and ethics statement

ICR strain mice were used in this study. All protocols were approved by the Animal Care and Use Committee of Sun Yat-Sen University, and performed according to the institutional Animal Care and Use Committee of Sun Yat-Sen University, People’s Republic of China. Old oocytes were collected from about 42-week-old reproductive aging female ICR mice and young oocytes were from 4 to 6 weeks female mice.

### Antibodies

Mouse monoclonal anti-α-tubulin-FITC antibody was purchased from Sigma Chemical Co. (Cat#:T6074); mouse polyclonal anti-HtrA2 antibody was purchased from Cell signaling (Cat#:2176); goat polyclonal anti-BubR1 antibodies were purchased from Abcam (Cat#:28193); FITC-conjugated goat anti-rabbit IgG purchased from Thermo Fisher Scientific; Mouse polyclonal anti-acetyl-tubulin (Lys-40) antibodies were purchased from Sigma (Cat#: T7451); other antibodies were purchased from Sigma (St. Louis, MO, USA).

### Oocyte collection and culture

Oocyte collection was performed on female ICR mice. To collect fully grown germinal vesicle oocytes, 4–6 weeks female mice were superovulated with 5 IU pregnant mare serum gonadotropin via intraperitoneal injection. After 48 h, cumulus-oocyte complexes were obtained through manually rupturing the antral ovarian follicles. Next, the cumulus cells were removed through mechanical pipetting in M2 medium (Sigma; Cat#: M7167). For in vitro maturation, oocytes were cultured in M2 medium for approximately 14 h under liquid mineral oil at 37 °C in a 5% CO_2_ atmosphere.

### Plasmid construction and mRNA synthesis

Total RNA was extracted from mouse GV oocytes using the Arcturus Pico Pure RNA Isolation Kit (Applied Biosystems, USA) and cDNA synthesis was performed using the QIA quick PCR Purification Kit (Qiagen, Germany). The cDNA fragment of HtrA2 was amplified using the following primers.

5′-GGTACCAGGAGAGTCGAGGCGGAGCT-3′.

5′- GAATTCCCGGTCCAGTCATTCATTCT-3′.

The PCR products were purified, digested with *KpnI* and *EcoRI* (NEB Inc., MA, USA), and cloned into the pcDNA3.1-EGFP vector. Western blotting was performed to verify the success of the overexpression. While, considering the EGFP was large, which might affect the expression of HtrA2. And the green fluorescence affected the detection of immunofluorescence. Therefore, we used *NheI* and *KpnI* to cut the EGFP tag before microinjection and carried out subsequent immunofluorescence experiments. For the synthesis of HtrA2 mRNA, plasmids were linearized using *XbaI*, and capped mRNAs were synthesized via in vitro transcription with T7 mMESSAGE mMACHINE (Thermo, USA) according to the manufacturer’s instructions. We constructed encoding frame shifted HtrA2 plasmid by site directed add ‘A’ caused premature termination according to the published protocol [24]. The mRNA was then purified using RNeasy Micro Kit (Qiagen). The synthesized mRNA was stored at − 80 °C.

The primers used were as follows.

5′- GTAAGCTTGGTACCAAGGAGAGTCGAGGCGGAGCTGA-3′.

5′- TCAGCTCCGCCTCGACTCTCCTTGGTACCAAGCTTAC-3′.

### HtrA2 knockdown and overexpression

Microinjections of HtrA2 siRNA or mRNA with a microinjector were used to knockdown or overexpress HtrA2 in GV oocytes, respectively. HtrA2 siRNA microinjection was used to knockdown endogenous HtrA2 in GV oocytes. The control group was injected with the negative control (Sangon Biotech, Shanghai, China).

The sequence information is listed below.

siRNA1#.

5′-CCAUCCCUUCUGAUCGCCUUATT-3′.

5′-UAAGGCGAUCAGAAGGGAUGGTT-3′.

siRNA2#.

5′-CAGUACAAUUUCAUCGCAGAUTT-3′.

5′-AUCUGCGAUGAAAUUGUACUGTT-3′.

siRNA- negative control.

5′-UUCUCCGAACGUGUCACGUTT-3′.

5′-ACGUGACACGUUCGGAGAATT-3′.

For overexpression experiments, approximately 10 pL mRNA solution (10 ng/μL) was injected into the GV oocyte cytoplasm. An equal amount of nuclease-free water was injected as a control. For knockdown experiments, HtrA2 siRNA was diluted with water to give a stock concentration of 1 mM and then a 2.5 μM siRNA solution was injected into the GV oocytes. A negative control was injected as a control. After injection, the oocytes were arrested at GV stage in M2 medium supplemented with 2.5 μM milrinone for 16 h. The role of the addition of milrinone could provide siRNA with a more time to degrade the mRNA of a single endogenous gene corresponding to its sequence. After several washes, oocytes were cultured in M2 medium at 37 °C in a humidified atmosphere of 5% CO_2._

### Quantitative real-time PCR (RT-PCR)

Quantitative RT-PCR was used to analyze the mRNA levels of HtrA2 and other deacetylases. Total RNA was extracted from 60 oocytes using a kit purchased from Invitrogen. cDNA was synthesized using the Prime Script RT Master Mix purchased from Takara. The samples were then quantified using ABI Step One Plus Real-Time PCR System. The fold change in relative gene expression was calculated using the 2^-ΔΔCt^ method. GAPDH was used as the housekeeping gene. Our data are expressed as fold-change relative to the control. Primers were designed using the Primer Bank website (pga.mgh.harvard.edu/primer bank/).

### Western blotting

GV oocytes were lysed in Laemmli (SDS-sample) buffer supplemented with protease inhibitor and boiled for approximately 5 min. Samples were separated via 15% SDS-PAGE, transferred to a PVDF membrane, and blocked in 5% low-fat dry milk diluted with TBST for 1 h. The cells were then incubated with primary antibodies (mouse anti-HtrA2 antibody, 1:1000; others, 1:3000) overnight at 4 °C. After several washes, membranes were incubated with secondary antibodies for 1 h at 37 °C. After multiple washes, the protein bands were detected using Bio-Rad Chemi Doc XRS +. Membranes were washed with a stripping buffer and incubated with an anti-actin antibody as a control.

### Immunofluorescence

The oocytes were fixed with 4% paraformaldehyde for 30 min and permeabilized with 0.5% Triton X-100 for 20 min. The oocytes were blocked with 1% BSA for 1 h. The samples were incubated with a primary antibody overnight at 4 °C. The next day, the oocytes were incubated for 1 h with a secondary antibody at room temperature. For F-actin staining, the samples were fixed with 3.7% paraformaldehyde for 5 min and then blocked with 1% BSA for 1 h. After incubation with FITC-conjugated phalloidin for 1 h, the DNA was counterstained with PI or Hoechst33342 for 10 min. Samples were washed several times, mounted on slides, and stained with 0.5 mg/mL DAPI in Vectashield mounting medium under a cover slip. The samples were analyzed using a laser scanning confocal microscope (Leica). The fluorescence intensity was measured using the ImageJ software.

### Chromosome spreading

Chromosome spreading was performed as previously described [35]. MII oocytes were exposed to Tyrode’s buffer (pH 2.5) for approximately 60 s to remove the zona pellucida. After recovery in M2 medium for approximately 10 min, the oocytes were transferred to 1% sodium citrate for 60 s, and the observed oocytes were slightly enlarged. The oocytes were then blown into a drop of 1% paraformaldehyde on a glass slide. The oocyte was slowly burst, which could not only separate chromosome, but also observed the membrane in the field of vision. After drying, oocytes chromosomes were stained with Hoechst 33342, and samples were observed under a laser scanning confocal microscope (Zeiss).

### Statistical analysis

Data are shown as mean ± SEM, unless otherwise indicated. Differences between two groups were analyzed by Student’s t test. Multiple comparisons between more than two groups were analyzed by ANOVA Tukey’s multiple comparison test. The analyzed were performed using GraphPad Prism 8 software. *p* < 0.05 was considered significant.

## Supplementary Information

**Additional file 1: Supplement Fig** **1.** HtrA2-KD affects mitochondria distribution in mouse oocyte meiosis. (**A**) Mitochondria dispersed from spindle periphery and cluster in the cytoplasm after HtrA2 siRNA injection. Scale bar: 25 μm (**B**) The percentage of abnormal mitochondrial distribution in HtrA2-KD group (*n* = 29) was significantly higher than that in control group (*n* = 25). Data are expressed as the mean ± SEM of three independent experiments. *, *p* < 0.05. HtrA2-KD, use siRNA-1.

**Additional file 2.**

## Data Availability

All data generated or analyzed during this study are available from the corresponding author on reasonable request.

## Abbreviations

HtrA2: High-temperature requirement protease A2
KD: knockdown
OE: overexpression
GV oocytes: germinal vesicle oocytes
MI: metaphase I
Pb1: first polar body
MII: metaphase II
SACs: spindle assembly checkpoints
ROS: reactive oxygen species
PMSG: Pregnant Mares Serum Gonadotropin
COCs: cumulus-oocyte complexes
GAPDH: glyceraldehydes-3-phosphate dehydrogenase
NC: negative control
PI: propidium iodide
DAPI: 6-diamino-2-phenylindole.
