# Supplementary figures and images for: Insufficient HtrA2 causes meiotic defects in aging germinal vesicle oocytes
Source: Reprod Biol Endocrinol. 2022 Dec 20;20:173. doi: 10.1186/s12958-022-01048-4 (PMC9764539; doi:10.1186/s12958-022-01048-4)

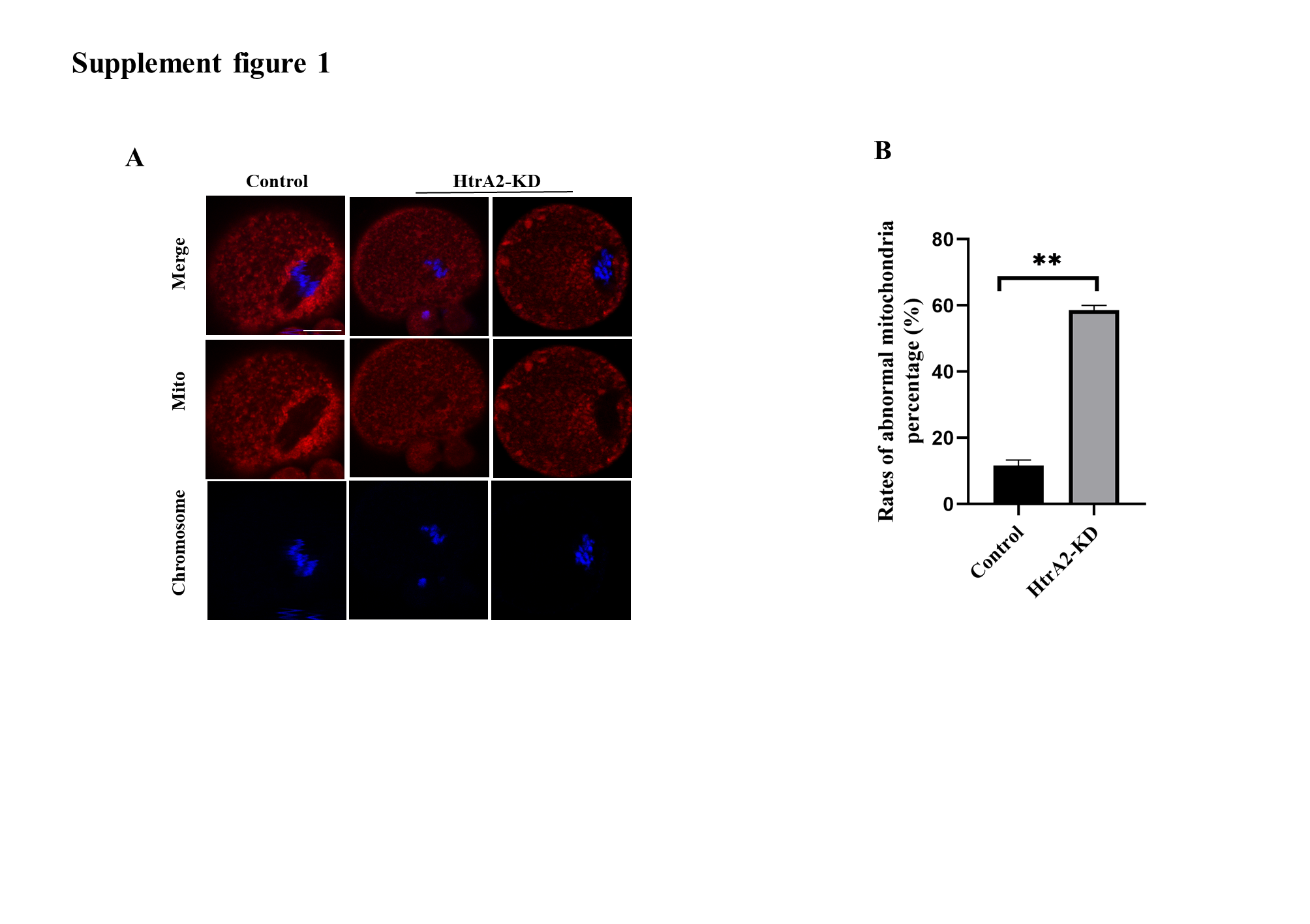

Supplement: Supplementary file 1 — Additional file 1: Supplement Fig 1. HtrA2-KD affects mitochondria distribution in mouse oocyte meiosis. (A) Mitochondria dispersed from spindle periphery and cluster in the cytoplasm after HtrA2 siRNA injection. Scale bar: 25 μm (B) The percentage of abnormal mitochondrial distribution in HtrA2-KD group (n = 29) was significantly higher than that in control group (n = 25). Data are expressed as the mean ± SEM of three independent experiments. *, p < 0.05. HtrA2-KD, use siRNA-1. [file 12958_2022_1048_MOESM1_ESM.tif]
