# Supplementary material for: Insufficient HtrA2 causes meiotic defects in aging germinal vesicle oocytes
Source: Reprod Biol Endocrinol. 2022 Dec 20;20:173. doi: 10.1186/s12958-022-01048-4 (PMC9764539; doi:10.1186/s12958-022-01048-4)
Supplement: Supplementary file 2 — Additional file 2. [file 12958_2022_1048_MOESM2_ESM.pptx]

## Slide 1
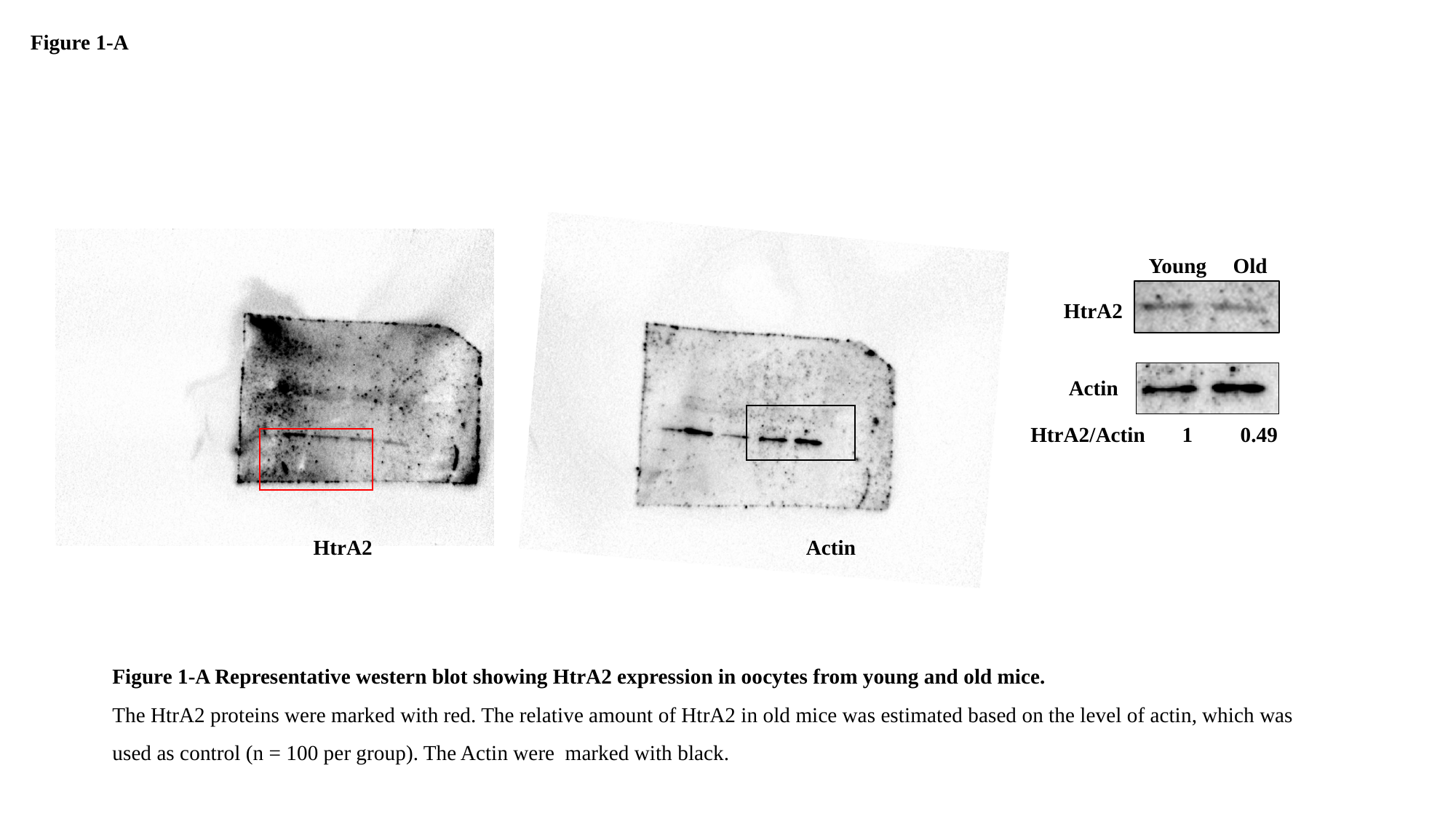

Figure 1-A
 Young Old
HtrA2
Actin
HtrA2/Actin 1 0.49
HtrA2
Actin
Figure 1-A Representative western blot showing HtrA2 expression in oocytes from young and old mice.
The HtrA2 proteins were marked with red. The relative amount of HtrA2 in old mice was estimated based on the level of actin, which was used as control (n = 100 per group). The Actin were marked with black.

## Slide 2
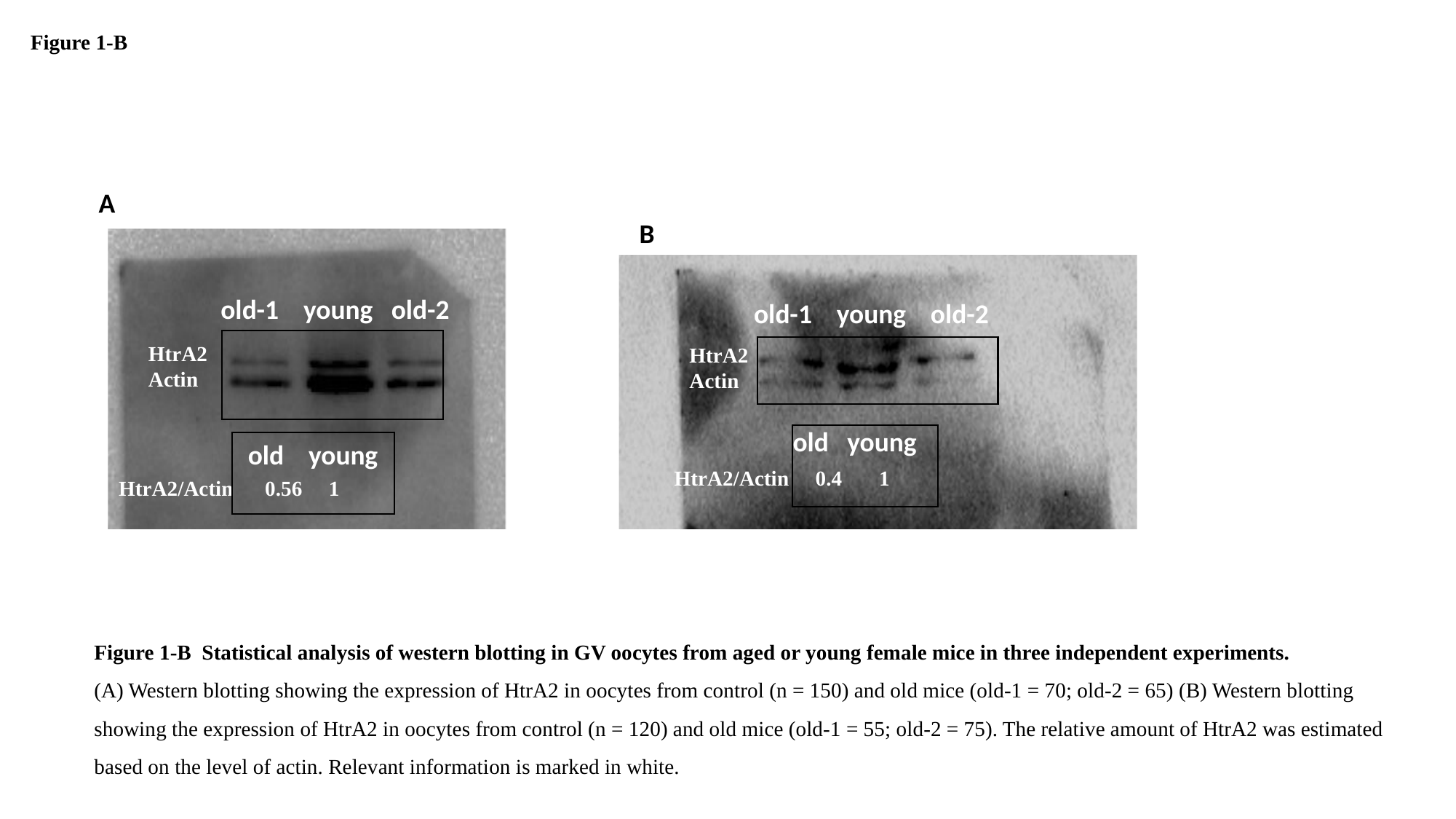

Figure 1-B
A
B
old-1 young old-2
HtrA2
Actin
old-1 young old-2
HtrA2
Actin
HtrA2/Actin 0.4 1
HtrA2/Actin 0.56 1
old young
old young
Figure 1-B Statistical analysis of western blotting in GV oocytes from aged or young female mice in three independent experiments.
(A) Western blotting showing the expression of HtrA2 in oocytes from control (n = 150) and old mice (old-1 = 70; old-2 = 65) (B) Western blotting showing the expression of HtrA2 in oocytes from control (n = 120) and old mice (old-1 = 55; old-2 = 75). The relative amount of HtrA2 was estimated based on the level of actin. Relevant information is marked in white.

## Slide 3
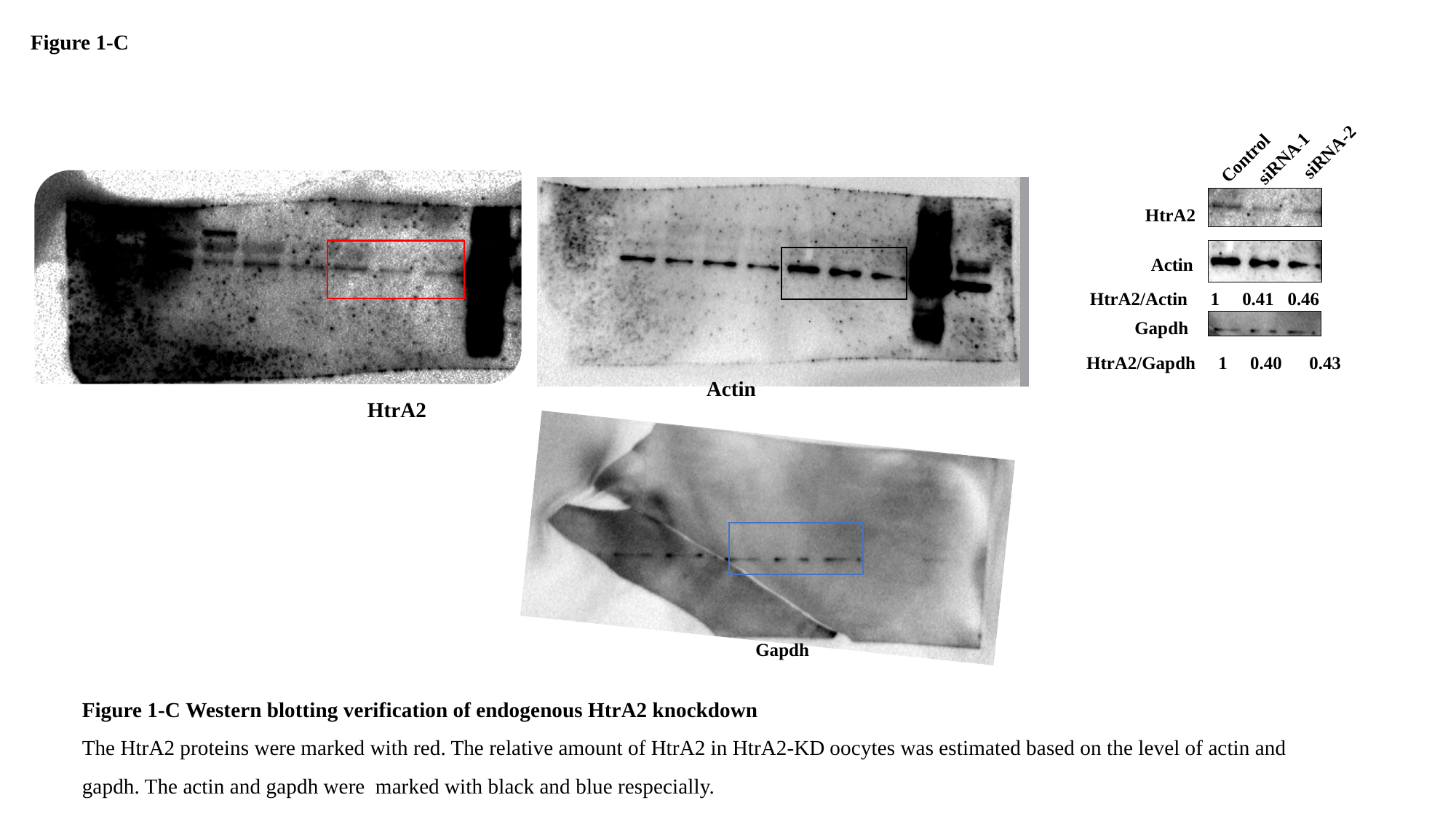

Figure 1-C
Control
 siRNA-1
 siRNA-2
HtrA2
Actin
Gapdh
 HtrA2/Gapdh 1 0.40 0.43
HtrA2/Actin 1 0.41 0.46
Actin
HtrA2
Gapdh
Figure 1-C Western blotting verification of endogenous HtrA2 knockdown
The HtrA2 proteins were marked with red. The relative amount of HtrA2 in HtrA2-KD oocytes was estimated based on the level of actin and gapdh. The actin and gapdh were marked with black and blue respecially.

## Slide 4
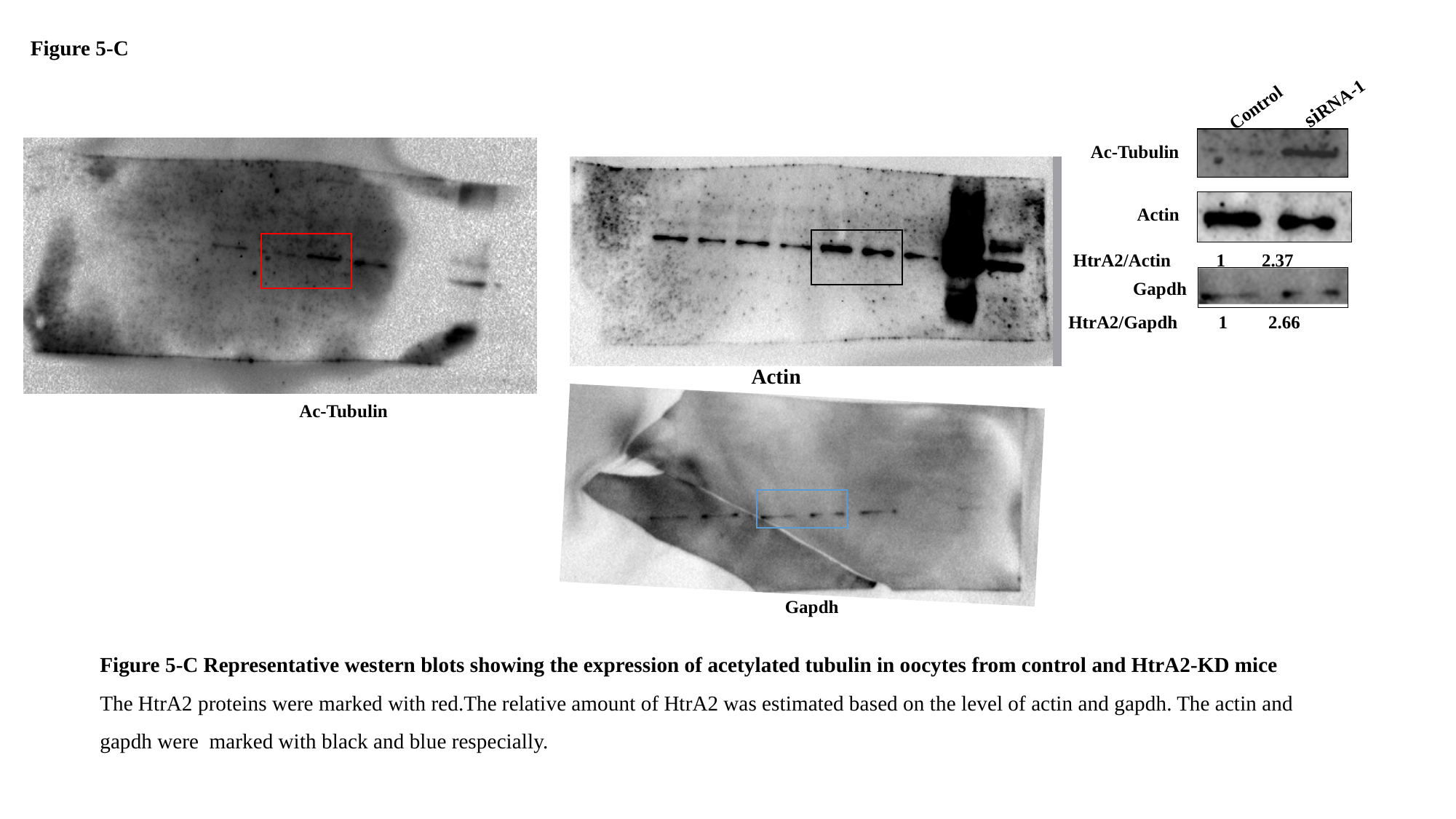

Figure 5-C
siRNA-1
Ac-Tubulin
HtrA2/Gapdh 1 2.66
 Control
Gapdh
Actin
HtrA2/Actin 1 2.37
Actin
Ac-Tubulin
Gapdh
Figure 5-C Representative western blots showing the expression of acetylated tubulin in oocytes from control and HtrA2-KD mice
The HtrA2 proteins were marked with red.The relative amount of HtrA2 was estimated based on the level of actin and gapdh. The actin and gapdh were marked with black and blue respecially.

## Slide 5
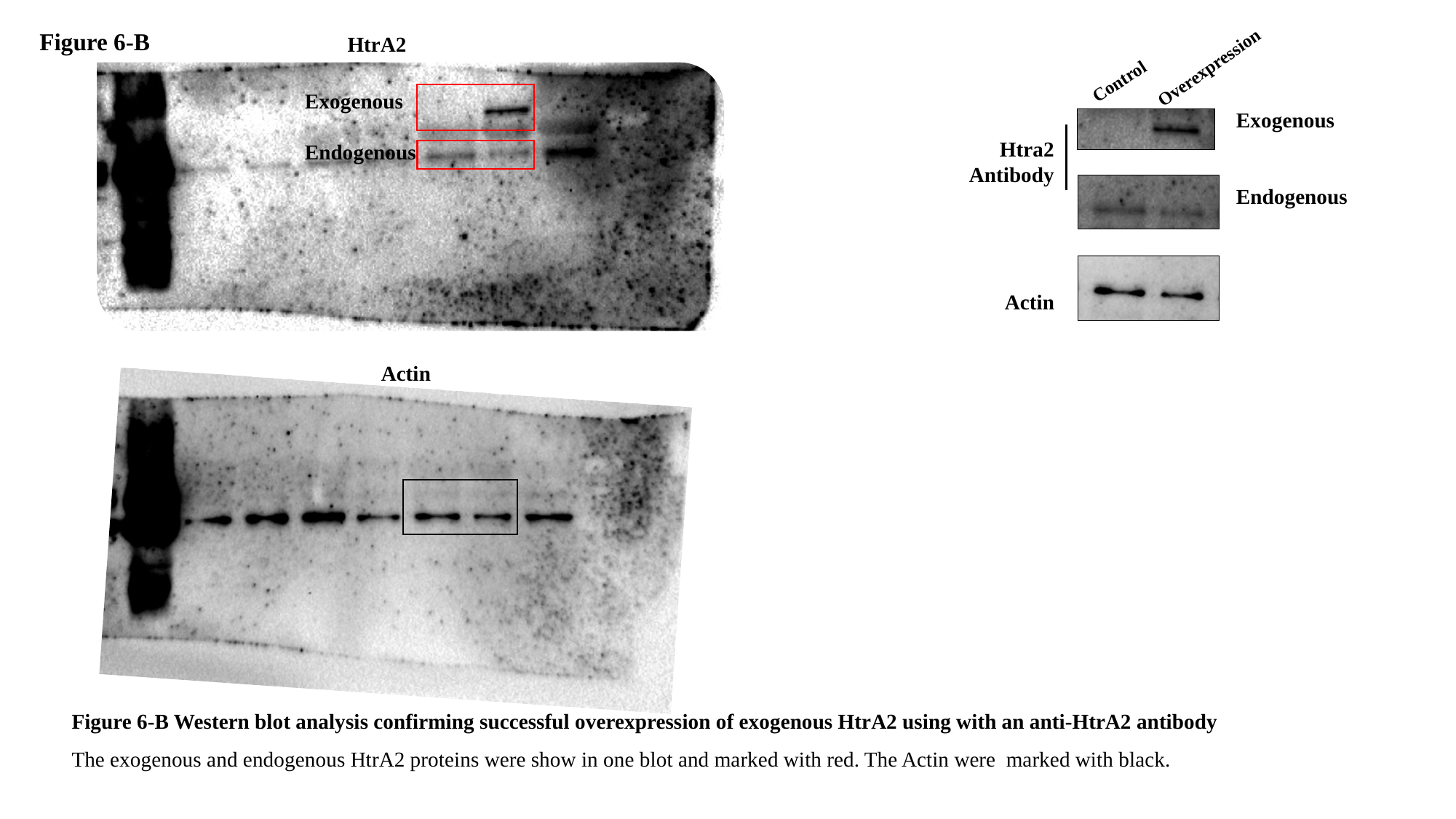

Figure 6-B
HtrA2
Actin
 Overexpression
 Control
Exogenous
Endogenous
Htra2 Antibody
Actin
Exogenous
Endogenous
Figure 6-B Western blot analysis confirming successful overexpression of exogenous HtrA2 using with an anti-HtrA2 antibody
The exogenous and endogenous HtrA2 proteins were show in one blot and marked with red. The Actin were marked with black.
